# Supplementary material for: The impact of cigarette smoke exposure, COPD, or asthma status on ABC transporter gene expression in human airway epithelial cells
Source: Sci Rep. 2019 Jan 17;9:153. doi: 10.1038/s41598-018-36248-9 (PMC6336805; doi:10.1038/s41598-018-36248-9)
Supplement: Supplementary file 1 — Supplement Tables 1-7 [file 41598_2018_36248_MOESM1_ESM.pdf]

The impact of cigarette smoke exposure, COPD, or asthma status on ABC transporter gene expression in human airway epithelial cells

Jennifer A. Aguiar, Andrea Tamminga, Briallen Lobb, Ryan D. Huff, Jenny P. Nguyen, Yechan Kim Anna Dvorkin-Gheva, Martin R. Stampfli, Andrew C. Doxey, Jeremy A. Hirota

Supplement Table 1: GEO Dataset Demographics

Comparison of Dataset Demographics										
GEO ID	GSE994	GSE4498	GSE11784	GSE11906	GSE37147	GSE4302	GSE63142	GSE67472	GSE76227
Airway Generation	Large airway (generation 2nd-3rd)	Small airway (generation 10th-12th)	Small airway (generation 10th-12th)	Trachea, large (generation 2nd-3rd), & small airway (generation 10th-12th)	Medium airway (generation 6th-8th)	Medium airway (generation 3rd-5th)	Medium airway (generation 3rd-5th)	Medium airway (generation 3rd-5th)	Medium airway (generation 3rd-5th)
Sample Type	Epithelial cells from brushings	Epithelial cells from brushings	Epithelial cells from brushings	Epithelial cells from brushings	Epithelial cells from brushings	Epithelial cells from brushings	Epithelial cells from brushings	Epithelial cells from brushings	Epithelial cells from brushings
Number and Classification of Patients	34 current smokers, 14 former smokers (average duration of smoking cessation was 10.49 years), and 23 never-smokers, with current and former smokers having >22 pack years smoking history with no reported COPD	10 individuals with >25 pack years smoking history with no reported COPD	72 individuals with >25 pack years smoking history with no reported COPD<br><br>36 individuals with >34 pack years smoking history with reported COPD, 72 individuals with >25 pack years smoking history with no reported COPD	17 trachea, 21 large airway, and 35 small airway from healthy individuals<br><br>54 individuals with >25 pack years smoking history with no reported COPD<br><br>20 individuals with >38 pack years smoking history with reported COPD, 54 individuals with >25 pack years smoking history with no reported COPD	69 current smokers and 82 former smokers (average duration of smoking cessation was 11.11 years) with >47 pack years smoking history with no reported COPD<br><br>87 individuals with >51 pack years smoking history with reported COPD, 151 individuals with >47 pack years smoking history with no reported COPD	28 healthy individuals and 42 asthmatics	26 healthy individuals, 59 mild asthmatics, 19 moderate asthmatics and 51 severe asthmatics	43 healthy individuals and 62 asthmatics	26 healthy individuals, 59 mild asthmatics, 19 moderate asthmatics and 51 severe asthmatics
Microarray Platform	Affymetrix Human Genome U133A	Affymetrix Human Genome U133 Plus 2	Affymetrix Human Genome U133 Plus 2	Affymetrix Human Genome U133 Plus 2	Affymetrix Human Gene 1 ST	Affymetrix Human Genome U133 Plus 2	Agilent 014850 Whole Human Genome Microarray 4x44K G4112F	Affymetrix Human Genome U133 Plus 2	Affymetrix HT HG U133 plus PM
Normalization Method	Mas5 normalization without log transformation	Mas5 normalization without log transformation	Mas5 normalization without log transformation	Mas5 normalization without log transformation	Robust Multi-array Average (RMA) with log transformation	Robust Multi-array Average (RMA) with log transformation	Cyclic LOESS	Robust Multi-array Average (RMA) with log transformation	Robust Multi-array Average (RMA) with log transformation

The impact of cigarette smoke exposure, COPD, or asthma status on ABC transporter gene expression in human airway epithelial cells

Jennifer A. Aguiar, Andrea Tamminga, Briallen Lobb, Ryan D. Huff, Jenny P. Nguyen, Yechan Kim
Anna Dvorkin-Gheva, Martin R. Stampfli, Andrew C. Doxey, Jeremy A. Hirota

Supplement Table 2: ABC Transporter Expression in Different Airway Generations

GSE11906 – Small vs Large Airway				
Probeset ID	Probeset Name	Raw p values	Benjamini Hochberg Adjustment	Log2 FC in Small Airway
230913_at	ABCG1	0.180	0.180	-0.894
202804_at	ABCC1	0.180	0.180	-0.874
1557374_at	ABCC9	0.180	0.180	-0.851
217504_at	ABCA6	0.180	0.180	-0.776
213485_s_at	ABCC10	0.180	0.180	-0.769
243951_at	ABCB1	0.180	0.180	-0.693
203196_at	ABCC4	0.180	0.180	-0.681
1555323_at	ABCB9	0.180	0.180	-0.631
206155_at	ABCC2	0.180	0.180	-0.614
203192_at	ABCB6	0.180	0.180	-0.613

GSE11906 – Large Airway vs Trachea				
Probeset ID	Probeset Name	Raw p values	Benjamini Hochberg Adjustment	Log2 FC in Large Airway
204719_at	ABCA8	0.053	0.079	0.963
1552590_a_at	ABCC12	0.053	0.079	0.854
220383_at	ABCG5	0.053	0.079	0.807
1570505_at	ABCB4	0.053	0.079	0.347
1554911_at	ABCC11	0.053	0.079	0.133
209735_at	ABCG2	0.074	0.110	-0.686
233371_at	ABCC13	0.074	0.110	0.423
240717_at	ABCB5	0.037	0.111	0.912
210245_at	ABCC8	0.101	0.152	0.389
231751_at	ABCG8	0.136	0.204	1.474

GSE11906 – Small Airway vs Trachea				
Probeset ID	Probeset Name	Raw p values	Benjamini Hochberg Adjustment	Log2 FC in Small Airway
202804_at	ABCC1	0.007	0.022	-0.983
203196_at	ABCC4	0.007	0.022	-0.916
225973_at	ABCB3	0.007	0.022	-0.902
230913_at	ABCG1	0.007	0.022	-0.866
1557374_at	ABCC9	0.007	0.022	-0.849
213485_s_at	ABCC10	0.007	0.022	-0.752
200045_at	ABCF1	0.007	0.022	-0.743
219577_s_at	ABCA7	0.007	0.022	-0.678
203192_at	ABCB6	0.007	0.022	-0.627
1554878_at	ABCD3	0.007	0.022	-0.573

The impact of cigarette smoke exposure, COPD, or asthma status on ABC transporter gene expression in human airway epithelial cells

Jennifer A. Aguiar, Andrea Tamminga, Briallen Lobb, Ryan D. Huff, Jenny P. Nguyen, Yechan Kim
Anna Dvorkin-Gheva, Martin R. Stampfli, Andrew C. Doxey, Jeremy A. Hirota

Supplement Table 3: Impact of Smoking Status on ABC Transporter Expression

**GSE11906 - Non-smokers vs Smokers**				
Probeset ID	Probeset Name	Raw p values	Benjamini Hochberg Adjustment	Log2 FC in Smokers
203192_at	**ABCB6**	1.58E-09	1.79E-07	1.194
208161_s_at	**ABCC3**	7.65E-08	4.32E-06	1.045
1553295_at	ABCA13	1.58E-06	4.45E-05	-1.045
1553604_at	**ABCA13**	1.20E-06	4.45E-05	-0.731
209620_s_at	ABCB7	5.58E-06	1.26E-04	-0.455
1553605_a_at	ABCA13	8.82E-06	1.58E-04	-0.739
202804_at	**ABCC1**	9.76E-06	1.58E-04	0.562
209641_s_at	ABCC3	5.51E-05	7.79E-04	1.172
230682_x_at	ABCC3	7.25E-05	9.11E-04	0.636
223320_s_at	ABCB10	1.19E-04	1.22E-03	-0.219

**GSE11784 - Non-smokers vs Smokers**				
Probeset ID	Probeset Name	Raw p values	Benjamini Hochberg Adjustment	Log2 FC in Smokers
203192_at	**ABCB6**	4.64E-10	5.24E-08	0.902
202804_at	**ABCC1**	2.75E-09	1.56E-07	0.624
208161_s_at	**ABCC3**	3.63E-06	1.37E-03	0.857
207622_s_at	ABCF2	1.83E-04	5.18E-03	-0.258
1553605_a_at	ABCA13	2.46E-04	5.55E-03	-0.785
230682_x_at	ABCC3	4.09E-04	7.71E-03	0.378
216066_at	ABCA1	2.84E-03	0.046	0.663
1553604_at	**ABCA13**	5.82E-03	0.072	-0.509
209620_s_at	ABCB7	5.34E-03	0.072	-0.212
211224_s_at	ABCB11	6.34E-03	0.072	0.390

**GSE4498 - Non-smokers vs Smokers**				
Probeset ID	Probeset Name	Raw p values	Benjamini Hochberg Adjustment	Log2 FC in Smokers
203192_at	**ABCB6**	1.39E-04	0.016	0.928
208161_s_at	**ABCC3**	6.00E-04	0.034	1.006
1553604_at	**ABCA13**	5.64E-03	0.213	-1.124
1553605_a_at	ABCA13	0.014	0.311	-1.022
215559_at	ABCC6	0.021	0.388	-0.322
1558460_at	ABCC5	0.030	0.405	-0.293
209994_s_at	ABCB1	0.030	0.405	0.271
1569072_s_at	ABCB5	0.036	0.405	0.204
202804_at	**ABCC1**	0.036	0.405	0.296
209641_s_at	ABCC3	0.043	0.437	0.377

The impact of cigarette smoke exposure, COPD, or asthma status on ABC transporter gene expression in human airway epithelial cells

Jennifer A. Aguiar, Andrea Tamminga, Briallen Lobb, Ryan D. Huff, Jenny P. Nguyen, Yechan Kim
Anna Dvorkin-Gheva, Martin R. Stampfli, Andrew C. Doxey, Jeremy A. Hirota

Supplement Table 4: Impact of Smoking Cessation on ABC Transporter Expression

**GSE37147 - Current vs Former Smokers**				
Probeset ID	Probeset Name	Raw p values	Benjamini Hochberg Adjustment	Log2 FC in Smokers
85320_at	ABCC11	3.89E-04	0.019	0.002
24_at	ABCC4	1.06E-03	0.026	-0.007
8714_at	**ABCC3**	2.79E-03	0.041	0.137
10058_at	**ABCB6**	3.31E-03	0.041	0.139
22_at	ABCB7	5.22E-03	0.045	-0.012
5244_at	ABCB4	5.47E-03	0.045	0.042
4363_at	**ABCC1**	6.89E-03	0.046	0.069
5825_at	ABCD3	7.47E-03	0.046	-0.011
154664_at	**ABCA13**	0.013	0.067	-0.058
64137_at	ABCG4	0.014	0.067	-0.010

**GSE994 - Current vs Former Smokers**				
Probeset ID	Probeset Name	Raw p values	Benjamini Hochberg Adjustment	Log2 FC in Current Smokers
202804_at	**ABCC1**	1.52E-04	2.29E-04	0.430
202805_s_at	ABCC1	5.45E-04	1.63E-03	0.564
203192_at	**ABCB6**	1.92E-03	5.75E-03	0.401
215703_at	CFTR	1.93E-03	5.80E-03	0.343
215873_x_at	ABCC10	1.96E-03	5.88E-03	-0.042
219577_s_at	ABCA7	0.014	0.021	-0.529
209641_s_at	**ABCC3**	9.77E-03	0.029	0.160
209993_at	ABCB1	0.042	0.064	-0.388
205043_at	CFTR	0.024	0.073	0.156
201873_s_at	ABCE1	0.026	0.078	0.119

GSE994 - Current vs Never Smokers				
Probeset ID	Probeset Name	Raw p values	Benjamini Hochberg Adjustment	Log2 FC in Current Smokers
202804_at	**ABCC1**	9.68E-05	2.90E-04	0.603
219577_s_at	ABCA7	8.10E-04	2.43E-03	-0.490
203981_s_at	ABCD4	7.02E-03	0.021	-0.317
203192_at	**ABCB6**	0.016	0.024	0.604
202394_s_at	ABCF3	0.020	0.061	-0.172
201873_s_at	ABCE1	0.053	0.080	0.268
203982_s_at	ABCD4	0.027	0.081	-0.510
210245_at	ABCC8	0.029	0.088	-0.193
215559_at	ABCC6	0.031	0.093	-0.503
202805_s_at	ABCC1	0.063	0.094	0.633

GSE994 - Former vs Never Smokers				
Probeset ID	Probeset Name	Raw p values	Benjamini Hochberg Adjustment	Log2 FC in Former Smokers
215703_at	CFTR	9.08E-03	0.014	0.899
205043_at	CFTR	0.034	0.051	-0.792
203196_at	ABCC4	0.023	0.068	-0.301
215465_at	ABCA12	0.055	0.082	0.475
202850_at	ABCD3	0.028	0.084	-0.128
214033_at	ABCC6	0.063	0.095	-0.294
202805_s_at	ABCC1	0.109	0.109	0.069
206155_at	ABCC2	0.041	0.122	-0.394
215873_x_at	ABCC10	0.158	0.158	-0.089
208561_at	ABCC9	0.060	0.180	-0.778

The impact of cigarette smoke exposure, COPD, or asthma status on ABC transporter gene expression in human airway epithelial cells

Jennifer A. Aguiar, Andrea Tamminga, Briallen Lobb, Ryan D. Huff, Jenny P. Nguyen, Yechan Kim
Anna Dvorkin-Gheva, Martin R. Stampfli, Andrew C. Doxey, Jeremy A. Hirota

Supplement Table 5: Association between COPD Status and ABC Transporter Expression

GSE11906 - Smoker without COPD vs Smoker with COPD				
Probeset ID	Probeset Name	Raw p values	Benjamini Hochberg Adjustment	Log2 FC in COPD
203505_at	ABCA1	1.91E-04	0.011	0.595
1555039_a_at	ABCC4	1.43E-04	0.011	-0.780
207583_at	ABCD2	2.59E-03	0.057	-0.493
1554878_a_at	ABCD3	2.39E-03	0.057	-0.551
204567_s_at	ABCG1	2.12E-03	0.057	0.385
232081_at	ABCG1	3.04E-03	0.057	0.457
202804_at	**ABCC1**	7.28E-03	0.091	0.262
1554918_a_at	ABCC4	7.28E-03	0.091	-0.494
1558460_at	ABCC5	6.52E-03	0.091	-0.283
204343_at	ABCA3	0.018	0.154	-0.485

GSE11784 - Smoker without COPD vs Smoker with COPD				
Probeset ID	Probeset Name	Raw p values	Benjamini Hochberg Adjustment	Log2 FC in COPD
213485_s_at	ABCC10	1.09E-03	0.123	0.338
226363_at	ABCC5	4.85E-03	0.274	-0.605
209994_s_at	ABCB1	0.028	0.344	-2.124
203191_at	ABCB6	0.015	0.344	0.745
223320_s_at	ABCB10	0.013	0.344	-0.440
202804_at	**ABCC1**	0.030	0.344	0.208
209380_s_at	ABCC5	0.018	0.344	-0.265
208480_s_at	ABCC6	0.030	0.344	-1.113
202850_at	ABCD3	0.028	0.344	-0.267
232081_at	ABCG1	0.030	0.344	0.429

GSE37147 - Smoker without COPD vs Smoker with COPD				
Probeset ID	Probeset Name	Raw p values	Benjamini Hochberg Adjustment	Log2 FC in COPD
225_at	ABCD2	8.37E-07	2.87E-05	-0.0716
10347_at	ABCA7	1.17E-06	2.87E-05	0.0321
26154_at	ABCA12	2.10E-05	2.62E-04	0.132
6891_at	TAP2	2.14E-05	2.62E-04	0.0387
6890_at	TAP1	5.72E-04	5.60E-03	0.0406
4363_at	**ABCC1**	8.29E-04	6.77E-03	0.0323
19_at	ABCA1	1.65E-03	0.012	0.0549
23456_at	ABCB10	3.49E-03	0.020	-0.00849
89845_at	ABCC10	3.60E-03	0.020	0.0128
9429_at	ABCG2	0.029	0.141	-0.0314

The impact of cigarette smoke exposure, COPD, or asthma status on ABC transporter gene expression in human airway epithelial cells

Jennifer A. Aguiar, Andrea Tamminga, Briallen Lobb, Ryan D. Huff, Jenny P. Nguyen, Yechan Kim
Anna Dvorkin-Gheva, Martin R. Stampfli, Andrew C. Doxey, Jeremy A. Hirota

Supplement Table 6: Association between Asthma Status and ABC Transporter Expression

**GSE67472 - No Asthma vs Asthma**				
Probeset ID	Probeset Name	Raw p values	Benjamini Hochberg Adjustment	Log2 FC in Asthmatics
154664_at	**ABCA13**	3.19E-07	1.50E-05	-0.438
1244_at	**ABCC2**	8.18E-06	1.92E-04	0.258
10057_at	ABCC5	1.81E-04	2.84E-03	-0.219
10060_at	**ABCC9**	4.39E-04	5.16E-03	-0.196
64240_at	ABCG5	9.43E-04	8.86E-03	-0.237
225_at	ABCD2	3.00E-03	0.023	-0.223
10351_at	ABCA8	9.59E-03	0.064	-0.161
10257_at	**ABCC4**	0.012	0.071	-0.146
8714_at	ABCC3	0.020	0.105	-0.099
10350_at	ABCA9	0.042	0.196	-0.118

**GSE4302 - No Asthma vs Asthma**				
Probeset ID	Probeset Name	Raw p values	Benjamini Hochberg Adjustment	Log2 FC in Asthmatics
206155_at	**ABCC2**	7.07E-05	7.99E-03	0.167
202805_s_at	**ABCC1**	6.88E-04	0.039	0.152
1553604_at	**ABCA13**	2.62E-03	0.079	-0.380
1553605_a_at	ABCA13	3.49E-03	0.079	-0.325
207623_at	ABCF2	3.22E-03	0.079	0.074
208462_s_at	**ABCC9**	5.00E-03	0.089	-0.167
210099_at	ABCA2	5.51E-03	0.089	0.129
1554918_a_at	**ABCC4**	0.010	0.128	-0.111
207583_at	ABCD2	0.010	0.128	-0.119
1557374_at	ABCC9	0.017	0.188	-0.506

The impact of cigarette smoke exposure, COPD, or asthma status on ABC transporter gene expression in human airway epithelial cells

Jennifer A. Aguiar, Andrea Tamminga, Briallen Lobb, Ryan D. Huff, Jenny P. Nguyen, Yechan Kim Anna Dvorkin-Gheva, Martin R. Stampfli, Andrew C. Doxey, Jeremy A. Hirota

Supplement Table 7: Association between Asthma Severity and ABC Transporter Expression

**GSE63142 - Mild/Moderate Asthma vs Severe Asthma**				
Probeset ID	Probeset Name	Raw p values	Benjamini Hochberg Adjustment	FC in Less Severe vs More Severe
A_23_P160940	ABCA4	4.75E-04	0.012	-0.143
A_23_P25559	**ABCC4**	8.43E-04	0.012	-0.118
A_24_P16913	ABCC4	5.43E-04	0.012	-0.081
A_24_P222291	ABCC4	7.27E-04	0.012	-0.062
A_23_P41380	ABCE1	3.65E-04	0.012	-0.162
A_23_P18713	ABCG2	1.01E-03	0.012	-0.197
A_23_P201918	ABCB10	1.63E-03	0.017	-0.097
A_23_P39481	ABCA7	5.30E-03	0.048	0.206
A_23_P219013	ABCC10	5.83E-03	0.048	0.081
A_24_P197196	ABCB6	8.98E-03	0.067	0.100

**GSE76227 - Moderate Asthma vs Severe Asthma**				
Probeset ID	Probeset Name	Raw p values	Benjamini Hochberg Adjustment	FC in Less Severe vs More Severe
1553604_PM_at	**ABCA13**	6.04E-04	0.065	-0.304
1553605_PM_a_at	ABCA13	2.12E-03	0.114	-0.251
1553295_PM_at	ABCA13	4.02E-03	0.145	-0.211
209246_PM_at	ABCF2	8.92E-03	0.241	0.183
214209_PM_s_at	ABCB9	0.019	0.414	0.182
241705_PM_at	ABCA5	0.041	0.427	-0.226
240717_PM_at	ABCB5	0.044	0.427	0.091
243167_PM_at	ABCB5	0.043	0.427	-0.146
1554918_PM_a_at	**ABCC4**	0.035	0.427	-0.077
205142_PM_x_at	ABCD1	0.027	0.427	0.108
